# Deadly River: Cholera and Cover-Up in Post-Earthquake Haiti

**DOI:** 10.3201/eid2211.161215

**Published:** 2016-11

**Authors:** J. Glenn Morris

**Affiliations:** Associate Editor, Emerging Infectious Diseases, Atlanta, Georgia, USA; University of Florida, Gainesville, Florida, USA

**Keywords:** cholera, bacteria, Haiti, book review

The massive magnitude 7.0 earthquake that hit Haiti on January 12, 2010, was followed 10 months later by onset of the largest cholera epidemic in recent history, with >768,831 cases and 9,113 deaths reported through the end of April 2016 (*1*). Although initial epidemic spikes were followed by a rapid decline in case numbers, cholera remains a critical public health problem for Haiti. Of immediate concern, the number of cases in the first 4 months of 2016 (close to 14,000 reported cases) exceeds the reported case numbers for the same period in 2014 and 2015 (*1*), consistent with the hypothesis that cholera in Haiti is becoming endemic, potentially with the establishment of environmental reservoirs (*2**,**3*).

There is a consensus that cholera was introduced into Haiti by Nepalese peacekeeping troops who were part of the United Nations Stabilization Mission in Haiti. Reaching this consensus, however, was not an easy process, given the associated political implications. Along the way, unfortunately, the political issues became intertwined in longstanding scientific controversies about basic transmission pathways for cholera. This resulted in scientific infighting among French and US research groups, personified on one side by Renaud Piarroux, based in Marseille, and on the other by Rita Colwell at the University of Maryland. In 1996, in an article in Science, Colwell articulated what has been termed the “cholera paradigm,” which places a strong emphasis on the role of the environment and environmental reservoirs in cholera persistence and transmission (*4*). Piarroux and colleagues, in contrast, have minimized the importance of the environment and environmental sources, both in Haiti and in Africa, and emphasized the critical role of controlling person-to-person transmission in eradicating cholera (*5**–**7*). Although the truth undoubtedly lies somewhere in the middle (*8*), the controversy has drawn in much of the cholera research community (including the Centers for Disease Control and Prevention and the World Health Organization) and has contributed to ongoing uncertainties about appropriate “on the ground” approaches to cholera control in Haiti in the face of increasing case numbers.

In this setting, Ralph Frerichs, professor emeritus of epidemiology at the University of California, Los Angeles, has published Deadly River: Cholera and Cover-Up in Post-Earthquake Haiti. In his preface, Dr. Frerichs clearly states that his goal was to highlight the work and scientific concepts put forward by Piarroux. In Dr. Frerichs’ words, “As I listened to his story unfolding in real time and heard more about the role of the United Nations and reputable scientists who were espousing suspect theories about the outbreak’s origins, I became convinced that the inside and ongoing story of what Piarroux had encountered in Haiti needed to be told.” As reflected in the title, Dr. Frerichs saw a cover-up and is seeking to make certain that Piarroux’s role and point of view (politically and scientifically) are clearly chronicled. 

As long as the reader is aware that there are other, well-supported, points of view from scientists with an equal dedication to control of cholera in Haiti, the book is of value in recording the events of this massive epidemic and the factors that led to its occurrence, from the vantage point of one of the major scientific investigators in Haiti at the time. However, the tone of the book and its tendency to see all events (and science) from the viewpoint of a single investigator/investigative group is not ideal and detracts from the impact that the work might otherwise have had. Science does have its controversies and infighting, and intermingling of scientific and political issues is inevitable; however, one might hope that our goal, as scientists, would be to resolve controversies with better science, while minimizing acrimony. Unfortunately, this book, with its focus on possible cover-ups and “suspect theories,” does not move us in this direction. 
